# Supplementary material for: Tipping the Balance: Vitamin D Inadequacy in Children Impacts the Major Gut Bacterial Phyla
Source: Biomedicines. 2022 Jan 26;10(2):278. doi: 10.3390/biomedicines10020278 (PMC8869474; doi:10.3390/biomedicines10020278)
Supplement: Supplementary file 1 [file biomedicines-10-00278-s001.zip › biomedicines-1531365-supplementary.pdf]

Supplementary Table S1. The Basic Characteristics of the Candidate Genes and SNPs selected

Genes	Full Name	Location	Selected SNPs
GC	Group-specific component	4q12-q13	14
VDR	Vitamin D receptor	12q13. 11	7
DNCR7/NADSYN1	7-Dehydrocholesterol reductase	11q13.4	3
CYP2R1	Viatmin D 25-Hydroxylase	11p15.2	7
CYP27B1	1-Alpha -Hydroxylase	12q13.1–q13.3	3
CYP24A1	Viatmin D 24-Hydroxylase	20q13	3

SNPs	Gene	Gene name
rs17219315	CYP24A1	Viatmin D 24-Hydroxylase
rs2244719	CYP24A1	Viatmin D 24-Hydroxylase
rs229624	CYP24A1	Viatmin D 24-Hydroxylase
rs2296241	CYP24A1	Viatmin D 24-Hydroxylase
rs2426496	CYP24A1	Viatmin D 24-Hydroxylase
rs4809960	CYP24A1	Viatmin D 24-Hydroxylase
rs6013897	CYP24A1	Viatmin D 24-Hydroxylase
rs10877012	CYP27B1	1-Alpha -Hydroxylase
rs4646536	CYP27B1	1-Alpha -Hydroxylase
rs703842	CYP27B1	1-Alpha -Hydroxylase
rs10500804	CYP2R1	Viatmin D 25-Hydroxylase
rs10741657	CYP2R1	Viatmin D 25-Hydroxylase
rs10766197	CYP2R1	Viatmin D 25-Hydroxylase
rs12794714	CYP2R1	Viatmin D 25-Hydroxylase
rs1562902	CYP2R1	Viatmin D 25-Hydroxylase
rs1993116	CYP2R1	Viatmin D 25-Hydroxylase
rs7116978	CYP2R1	Viatmin D 25-Hydroxylase
rs11234027	DHCR7/NADSYN1	7-Dehydrocholesterol reductase
rs12785878	DHCR7/NADSYN1	7-Dehydrocholesterol reductase
rs3829251	DNCR7/NADSYN1	7-Dehydrocholesterol reductase
rs1155563	GC	Group-specific component
rs12512631	GC	Group-specific component
rs16846876	GC	Group-specific component
rs17467825	GC	Group-specific component
rs222020	GC	Group-specific component
rs2282679	GC	Group-specific component
rs2298849	GC	Group-specific component
rs2298850	GC	Group-specific component
rs2882679	GC	Group-specific component
rs3755967	GC	Group-specific component
rs4588	GC	Group-specific component
rs7041	GC	Group-specific component
rs10783219	VDR	Vitamin D receptor
rs4516035	VDR	Vitamin D receptor
rs7139166	VDR	Vitamin D receptor
rs731236	VDR	Vitamin D receptor
rs757343	VDR	Vitamin D receptor

Supplementary Table S2. SNPs ID and related sequences.

Target Name	Sequence
rs2298849	ATTCTTTAATGTTCACAAGGGACATCTGCATTTATCCTGCTTTGTAAAATTAAGGTTTTCATTTAGTGCTGTCAGTTAACAGCCTCACCTAATTCGTACA[C/T]TATTATTCATAATAGTAATGTGTTTTGCCAGTGGAATAAAATGTGTGTTCCCAAGAATTATCAGTTATTAACTGTAACATGAATAACCTTACTCTTGACT
rs12512631	TACTACTATTGTTAATTGCAAACTAGAATTTTCCAGAATGCTTTAGCTATGAAACTAGTAGCCTTGTGGTGGTGGTTGTCATGGAAGAAACAATTAGATG[C/T]TTTCTTGGCCTAATAGAGAGAAAAGAATATTTCAGGTCCTGGAAAAAATGGACAAGGATAAAATAGCCTCTGGGAAAGAGAATCTTTTGACCTGATTATC
rs16846876	TTTTCGTTTGTGGATTTTGTTGTATTTTCGCAACTTTAATGCCTTGTCAGTTTTAGAATTGTAAATATATTATCCCTACCTGCACATGTCTGTGAACTTT[A/T]TTCATGATGTCTTTTGTTGAACAGAACTCCTAAACTTGATAATAAAATTMATCAATATTTTGCCTTATGATTTGTCCTCTTTGAATTTTTCTTAGGTAGG
rs17467825	AAATGGAACAAATGACATCATTATGCATATTAGATCTAACATTAAAACTCTTTTACTAAAATTACAATATTTCTGTCAGCGATTCTTAATATAAGAAAAA[A/G]TGGTGAAATGTGTTTAGAGTGTGCTGGAAAATATATCCATGGGAACTATGAAAACCCTACTTCAGTGAATTGTATATTTATTGACTACTTTGTGACAAWA
rs3755967	TGATGAAGGCCTACCAATGTTCAATTATTTCAGGAATAGTTACATGGCTTTGAAAAAGCAAATTATACCTTGTTCACAAAGTTATAGGTCTGAGGTACTT[A/G]ATATCCAGAAATAATGAGACCACAACTTTACCACTTATCTTAGGTTTCCAGAACACAACCAACTCATTGCAGTTCCTGGGTTCCAGGGAGAATTTTGTAG
rs2298850	AAAAGACCCATTTGCCTACAGAAAAACCTATAGTTACAAAATTAGACTTGAAATCTTCTTTTCTCTTTCTACCATCATTCAGGCAGGGCATCCCTTTGGG[C/G]TCTTCTGAGAACTCATTACTATGCTTCCTTTAAAATGTTTCAACAGACTGCTAGTGAGCATCAGATATGATTTCCCAAAGTATTCCAAAAGTCATTGTAA
rs731236	GGTGGGATTGAGCAGTGAGGKGCCCAGCTGAGAGCTCCTGTGCCTTCTTCTCTATCCCCGTGCCCACAGATCGTCCTGGGRTGCAGGACGCCGCGCTGAT[C/T]GWGGCCATCCAGGACCGCCTGTCCAACACACTGCAGACGTACATCCGSTGCCGCCACCCGCCCCCGGGCAGCCACCTGCTCTATGCCAAGATGATCCAGA
rs10783219	TGCCTACCCTTGCCTTCCTGGCTGCTTACCTGCTTTACAGCCAGACTTTCTGTGGGATAGTGTGGTCCCCAGAGGTCTGGGAAGCTGGATGAGCAAAATG[A/T]GGAGGCTGTAGATATGGAGGAAGAGGGAAGAGGAAACGTGAGGCCCAGTCTTGGCTCCACTATGAACCCCAATTTCCCCAGGGGAAGCTGAAGAGGAGTT
rs7139166	AGAAACAGATGAAGTGCCCCTTTAGAGGTCCTGCCAGTCTGATGGATGGATTCTCTACCTGTAATGAAAGTAATAGGAAGGATCCCCTTGCCCAAAGCAT[C/G]GTGGGAAAGCTAGCGGTGATTCTTGTGGGAAGAAAAGCATAAGAGGAAATTTCCAACCACCAATACCTTGGGACATTCTCTGCAAGTCCCATGAGCCTCT
rs4516035	GGAGAATGTATAAATAAATAGTAATTACAACATAATCTTCTGGAATAGAAATGCTCACAAAATCATCCAGCAGTCGATGACCTCCTTTAGCCAGGGAAGA[C/T]ATTGCTATTCGCCTCTTACAGAGGAGGAAACCATGGCTCAGAGGGACAAGGTGAAAGGGTGGGAAATTGAGGCATAGAAAGTTTAAGTGATATGCCCAAC
rs757343	AGGTTTCTTGCGGGCAGGGTACAAAACTTTGGAGCCTGAGAGATGGTTCTGCCTATATAGTTTACCTGATTGATTTTGGAGGCAATGTGCAGTGACCCTT[A/G]ACCTCTTCCGCTGGTTAGAGGTGAGAAGAGGGAGAAAAGGCCGAAGAGGAAGTTATTGTGACCTTGGGGACATGATGTCGGTGATGAGGTCCAAAGAGGG
rs703842	AAGAATACAAGATCCCGTCCTCCAGGCAGTGACCTCCCAAACCAGCCTGCCTGGTCACTGACTGCTTACTCTACCTTAGCTGGACCTCGTCTCCCAGGGA[C/T]TAGAGAAAAGAGCAGGAGTCCTGGGTCTTCCCAGTTGAGACTGCTGGAGCTGAGACACAGTACTCTCTTAAAGAAGGTGGGGAGCTGCCCAGGGCAGAAC
rs4646536	CAGGGAGATAGCAGAGAAAAATGGCCCTCTACTCCTGGCCAAAAAGGGTTTGGAAGTTGGAAACAATGAGAAGGGGGCTGCAGCCCCTAGCCTCATCTTG[C/T]TGTCTCCATTTTGTGCTTTGCAACCTAGACTGTACYCTGTGGTACCTGGAAATTCTSRTGTCCCAGACAAAGACATTCATGTGGGTGACTATATTATCCC
rs10877012	AGCCCGGGAGGTCAAGGCTGCAGTGAGTTATGATTGTACCACTGCACTCCAGCCTAGGCAACAGAGAGAGGGCCTGTCTCTAAAAAAAATTAAAATAAAA[G/T]AATCTCCCACAGTTTACCTCTCTGCTCCTTACTCCCTCATTCCCCACTGCAACTCTATCAGGCTCTGTGCCAGTCAGCTAGCTCTGAAGTTCTGGAATTG

rs1562902	CTACAAGTGTGCATAACATAGGAAATGTGATCAGTCTTGGAGGTCAGTAATTGTAGTCTGCACCAAACAACCAGCTTAT<br>ATCCAGGGACTCCCCCATGAT[C/T]GTGTTCATGGAAGATGTTACTACAATTCTTCCCTATCAACTGGTCTCATATGGAC<br>ACTGAGTGAAGGGAAAGGAAAGTAACAAAATCCTCTRTTTAAAAT
rs10500804	CATGGCACTTATTTAACACTATCTTGAACCAGAAACATTCATATTCTTGTCTTCTGCCTGCATGCTAAATTACAAACTCCTT<br>AGGAGCAGTGATTTCATC[G/T]TTTTCATCTTTGAATCCTTACACAGAATCTCATTCAGTGCTGTGCATAAAGTTCTCAAT<br>AAATGCTGTAAAAATAAATTAATACGCTGTCAATTCTACTT
rs10741657	AGAGCAATGACATGGAAATACAATAATTTTCAATAATCAGAAGCAAACAAAAAGTGCGTTATAGAAAACGCCTGGTGG<br>TTGGGGAGATACTTTAGCAGGC[A/G]AGGGCTGTCAAGGAAAGTCTTATTAGGAAGATGACATTGGAGCTGAGATCAA<br>CTGCTAAAGAAGTGTATTTGTTTCCTACTACTGTTGTAATAAATTACC
rs10766197	AATAGCAGTCCCTTTAATAGAAGGCTTTTGGCTGTCTCCTTCAAAAACCATAATCTCTGTACAATTTGGAACACTCCAGTT<br>AATTAGAGATCTTTAAACT[A/G]TGCCAAGATACAGAAAGGACCAAGCTTTAAAAAATATTGTCTTCCACATGGATTGG<br>TCTAAAAGCAGTATTTTAACCTAAGCTYTGAAATACAGTTTTAC
rs12794714	AGCCCGGGCCAGCCTGGCGGCCCTCCCTGCCCGGGGCCCGTCGGGGCTGTACCTCTCCGTACACCTGGCTCTGCTTTCT<br>CATGTAGACATGGGGAAGCTC[A/G]GATGAGGCTGCCAGGGAATAGATGTTGCCGATAAATGGCAGCCCCGGCGGCC<br>CCGGGGGGAAGCCCATCGGCCGCCTCTGCTTCAGCAGCTGGCGGACCC
rs7116978	TTAAAATTATTTCATAGTTGTATAGTCTTATTCACTTTAATTTTTAAATAAAATTCATTTAAGTGCTTAAGTCACCTTTTAT<br>AGGTAAAAGATTATCTAA[C/T]ACAATACTTTGAATGTGTATTCCTTAAAGACTTCTATTTTGAAGTCTCTCAAAAAAATG<br>CCATATGAATGGGGCTCTAGACTTCCTATTAAGTTTCAATC
rs1993116	TTATTGAGAACTTTATGCACAGCACTGAATGAGATTCTGTGTAAGGATTCAAAGATGAAAAMGATGAAATCACTGCTCC<br>TAAGGAGTTTGTAATTTAGCA[C/T]GCAGGCAGAAGACAAGAATATGAATGTTTCTGGTTCAAGATAGTGTTAAATAAG<br>TGCCATGGGAGCTTCTAAGCTCCCATGTGTGGTTCAAAGCAGAGAG
rs3829251	TGTGCTCACCAGGGTGTGCAGCAGGTCCTGGGAGTTGGCTGCAGGCTGGCTCCTACCCCTCCTCCTTTCTCTTCCCCTGC<br>CCTCACTGCCTCTTCCACGG[A/G]CAGTTCTCTATGTGAGGACAGTGGAAGGAGGGCACCTTTCCRGAGAAGRAAGACT<br>GGGTTCTATCTAGAATATTCCATCCATAATGGCTCCTAGGAGAAA
rs12785878	TGTTCTTTAATTTGTCTTCATTTGATTGATTCTTTAAGAAATTCTTCACCTAAGTGCCAAGGGATCTAGGGTTCTGGGCTG<br>TCTGATATCACAAAGCTTC[G/T]ATCCTCTCCTGGCCCCGTGGCCGGATCTTCTCCTGGGCTGGACTCAAGGCCTGATCG<br>TCTCGTGAGCCTGACCTCTCCTGGGCTGGTCCGGGGGCCTGAT
rs11234027	CTGGTGACATGTTGCTGTATTCTTGGGTGAGAATGCTRAGGACATGCTGCTGTATTCTTGGGCGAGTACATTGGTGACA<br>TGCTGCTGTGTTCTTAGGTGA[A/G]TACACTGGTGAAACAATGCCCAATCKCACGGCCAAGAGWTTTGAGGAGCAGGG<br>ACATCTGGTAAGCAGACTTCAGTTTCTTTTCAGTAATGTGCCCCTCT
rs2244719	AAGCCATTCACCAGAGGAAGAGAAGGGAAGGGAATAATTATGTTCTAAACACCCATTACACGCCAGGTACTGGGCTAG<br>CACTTCATATAGGGTTTTTTTT[C/T]CTTCCATTTCATCATTTTATTTAGAAGGAACCTTTTTACAAATAAAATTCTCAACTC<br>TTCATTTCAAAACTTTTTACCTAGAGAAAAGTTGAAGGAATGG
rs2296241	ACGACAACTTACTTTCAAACGACCATTTGTTCAGTTCGCTGTACAAGTCTTCAACGTGGCCTCTTTCATCACAGAGCTCAT<br>CTATTCTGCCCATAAAATC[A/G]GCCAAGACCTTCAAAGAAAACAACCGCAAAAGACACATTTTAAAGCCGTTGAAGGA<br>AAACAAAACTTTAAAGCTCACTGAGCTAACTTCAGGTCTTGCTA
rs2426496	GCTTGGGCTGAAACCATGGCTCCACCACTCATTAGCTGTGTGATCTTGGGCAAGTTATTTAACTTCTCTGAGTCTAGTTT<br>CCTTAATGGTAAAATTGGAG[G/T]TTACTATACCTGTGTCTCAGGAAGGTCAAGATAAATACTAAAAATATTAGACAAA<br>ACACGATTGTCTTCACTAAAATCTGTTTGGCCTCTGTACTTGCTT
rs4809960	GGCATAACTTAAGCACCTAAAAGAAGCATTAAAAGGGCTGCTCTGGCCTTTCCCTAGGCAGCAATAATGCCTGTTTACA<br>AAAGAGTTGTCAAAAGAGCCA[C/T]GTTTTATCCGCAAAATCACCTGCAAAATCAGTGAGCAAGTCTGTGACGACAACT<br>TACTTTCAAACGACCATTTGTTCAGTTCGCTGTACAAGTCTTCAAC
rs4588	AAATTTGTATAAAATAAATACATGTAGTAAGACCTTACATTTAAATGGTTTTTCAGACTGGCAGAGCGACTAAAAGCAA<br>AATTGCCTGAKGCCACACCCA[A/C]GGAACTGGCAAAGCTGGTTAACAAGCACTCAGACTTTGCCTCCAACTGCTGTTCC<br>ATAAACTCACCTCCTCTTTACTGTGATTCAGAGGTAGGAAAATGT

rs17219315	TTAGCATCAGAATATTTGGGATAAAATAGTGATCATCTTTTGACAATAGTTTGACAATAGGATGAAAAAGCACCTTTCCTCCTAGTCAAAGATTGCACCA[A/G]AAAGTTGAAGTCCAGATAATTTGGGTTCAGGGATTTTGAGGTGTCCTTGCAGGGAGTGGGGCTGGGAGGCCTGGGGACAATTCCTAGCGGTAAAAGGGGG
rs6013897	CTCTGCCACTTACCTACTGTGTATCTTTGGGTAGGTTACTTCTGCCTCTGTCTTGGTTTCCTCATCTGTAAAAGGGGGATAATGAAAGTACCTACTTCAG[A/T]GGATGATTACACTTCCTTCACATGCATTTACGAGTTTTCTGAACATATAATCCTGTTGAGATCCATGTGGACTTGATGATTCTAATTATTTGTCATGCAT
rs7041	TTTTGCATTAGAAATTTGTATAAAATAAATACATGTAGTAAGACCTTACATTTAAATGGTTTTTCAGACTGGCAGAGCGACTAAAAGCAAAATTGCCTGA[G/T]GCCACACCCAMGGAACTGGCAAAGCTGGTTAACAAGCACTCAGACTTTGCCTCCAACTGCTGTTCCATAAACTCACCTCCTCTTTACTGTGATTCAGAGG
rs229624	CATGCCTCCTCTTCATGTCGTTGATGTACCAGGCCACACAGGTCATGGTGTCAATGGCATCCTCCACCACCTCAAAKCCATCCTCTTCTTCATCAAAATG[C/T]TTGGCAATTTCCTGCGCAGAAGATAGACGCTTAGGTGCCCCTGACCCGAAGGCCCTGCCCTCCCCAGGCCCTAGGTGCTTGGGAGGACCGAGGGGCTACC
rs222020	GAATAACAAGGGAGAGAGATCACAGAAGAACACAGTAAGAGAAAACGGCAGCTTTACACATCTTGGTAACCAGAGGAGACAACCTTGGCTCACCATGCTG[C/T]CCATTGAATTTTTTGNCCATTGAGTTGAGTTTTTCCTGCTGCTATCTCTCTTACTAGCTTCTCCTTTCCTGTGTTAGCCCTGGAGGAGGTAGAAGCAGCC
rs2882679	TGATTTAGGACCATACCACGGAGAGACACAAAGTGGTACCAGAGACCAGCTCATTAAGTTTACCATATACAACTATGGTAATTTGTTTTTAAAAGATATT[C/T]TAAAACAGGTAAGTCAAAGTTTCACATATGGAAATATGTCCAAATTAAAATGTGCTTGTCTTCCTGAAGAGTCTGGGTCATTCATTAAAAAAAAAAAAAA
rs1155563	GAACTGATAATATCAAAATAACAAATTTACTTTCGTGTAGTAAAATTTGAAGACTGAAATGGTTATTCTAAGACTGTGCTCTTGCTATTGTANTTTTTAA[C/T]AGATAAAAAAATCACAGTTATTTCCATATAAAAATATTTTCACTCATAAATGGGAGTCGAACAGTGAGAACACATRGACACAGGGAGGGGAACATCACAC
rs2282679	TTCTGACCTTGTGATCCACCCACCTCGGCCTCCCAAAGTGCTGGGATTACAGGCGTGAGCCACCATGCCCAGCAAATCTCTGTCTCTTAATTATCTCACA[A/C]AGCCAGGTATTTTTTATTGTTAGCTTTGGAAGCAAGTAGTAGTCCCTCTGGGTTGCCAAAAACATAAATGAATTGGCTCACAGTGTAGAATCAGATTGGG

Supplementary Table S3. Allele frequencies of the SNPs assessed in this study. HWE: Hardy-Weinberg equilibrium; NA: not available. All alleles in the table below are reported in the Forward orientation.

SNP ID	Gene	Chr.	Allele	Current study cohort (N=94)	HWE P-value
rs10783219	VDR	12	Ref (T)	0.31	0.9
			Alt (A)	0.69	
rs7139166	VDR	12	Ref (C)	0.66	0.24
			Alt (G)	0.34	
rs731236	VDR	12	Ref (A)	0.51	0.31
			Alt (G)	0.49	
rs4516035	VDR	12	Ref (T)	0.66	0.24
			Alt (C)	0.34	
rs10741657	CYP2R1	11	Ref (A)	0.28	0.82
			Alt (G)	0.72	
rs10766197	CYP2R1	11	Ref (G)	0.6	0.08
			Alt (A)	0.4	
rs12794714	CYP2R1	11	Ref (G)	0.61	0.06
			Alt (A)	0.39	
rs1562902	CYP2R1	11	Ref (C)	0.41	0.65
			Alt (T)	0.59	
rs10500804	CYP2R1	11	Ref (T)	0.61	0.07
			Alt (G)	0.39	
rs1993116	CYP2R1	11	Ref (A)	0.29	0.56
			Alt (G)	0.71	
rs7116978	CYP2R1	11	Ref (T)	0.26	0.84
			Alt (C)	0.74	
**rs10877012**	CYP27B1	12	Ref (G)	0.8	**0.04***
			Alt (T)	0.2	
rs4646536	CYP27B1	12	Ref (A)	0.76	0.15
			Alt (G)	0.24	
rs703842	CYP27B1	12	Ref (A)	0.73	0.11
			Alt (G)	0.27	
rs11234027	DHCR7/NADSYN1	11	Ref (G)	0.7	0.09
			Alt (A)	0.3	
rs12785878	DHCR7/NADSYN1	11	Ref (G)	0.72	0.24
			Alt (T)	0.28	
rs3829251	DNCR7/NADSYN1	11	Ref (G)	0.69	0.07
			Alt (A)	0.31	
rs1155563	GC	4	Ref (T)	0.8	0.36
			Alt (C)	0.2	
rs12512631	GC	4	Ref (T)	0.64	0.17
			Alt (C)	0.36	
rs16846876	GC	4	Ref (A)	0.76	0.73
			Alt (T)	0.24	
rs17467825	GC	4	Ref (A)	0.8	0.75
			Alt (G)	0.2	
rs222020	GC	4	Ref (C)	0.2	0.47
			Alt (T)	0.8	
rs2282679	GC	4	Ref (T)	0.79	0.2
			Alt (G)	0.21	
rs2298849	GC	4	Ref (A)	0.73	0.13
			Alt (G)	0.27	
rs2298850	GC	4	Ref (G)	0.22	0.68
			Alt (C)	0.78	

**rs2882679**	GC	4	Ref (C)	1	**NA**
			Alt (T)	0	
rs3755967	GC	4	Ref (C)	0.79	0.19
			Alt (T)	0.21	
rs4588	GC	4	Ref (G)	0.77	0.59
			Alt (T)	0.23	
**rs7041**	GC	4	Ref (A)	0.56	**0.05***
			Alt (C)	0.44	
rs17219315	CYP24A1	20	Ref (A)	0.98	0.87
			Alt (G)	0.02	
rs2244719	CYP24A1	20	Ref (C)	0.37	0.46
			Alt (T)	0.63	
rs229624	CYP24A1	20	Ref (G)	0.79	0.44
			Alt (A)	0.21	
rs2296241	CYP24A1	20	Ref (G)	0.43	0.11
			Alt (A)	0.57	
rs2426496	CYP24A1	20	Ref (T)	0.47	0.16
			Alt (G)	0.53	
rs4809960	CYP24A1	20	Ref (T)	0.72	0.12
			Alt (C)	0.28	
rs6013897	CYP24A1	20	Ref (T)	0.7	0.78
			Alt (A)	0.3	

Supplementary Table S4. Association of genotypes with D/ND and SD/MD/ND (Chi-square test) and with vitamin D level (ANOVA)

**SNP ID**	**Gene**	**Chr.**	**P-value (D/ND)**	**P-value vitamin D level (ANOVA)**
rs10783219	VDR	12	P = 0.8447	P = 0.9100
rs7139166	VDR	12	P = 0.6943	P = 0.4528
rs731236	VDR	12	P = 0.1878	P = 0.3048
rs4516035	VDR	12	P = 0.6943	P = 0.4528
rs10741657	CYP2R1	11	P = 0.5257	P = 0.3263
rs10766197	CYP2R1	11	P = 0.9394	P = 0.7176
rs12794714	CYP2R1	11	P = 0.8940	P = 0.8546
rs1562902	CYP2R1	11	P = 0.5215	P = 0.8665
rs10500804	CYP2R1	11	P = 0.9421	P = 0.8547
rs1993116	CYP2R1	11	P = 0.5257	P = 0.3263

rs7116978	CYP2R1	11	P = 0.9656	P = 0.6342
rs4646536	CYP27B1	12	P = 0.6064	P = 0.6646
rs703842	CYP27B1	12	P = 0.8409	P = 0.6667
rs11234027	DHCR7/NADSYN1	11	P = 0.7029	P = 0.8015
rs12785878	DHCR7/NADSYN1	11	P = 0.3467	P = 0.3792
rs3829251	DNCR7/NADSYN1	11	P = 0.5888	P = 0.3886
rs1155563	GC	4	P = 0.3683	P = 0.9884
rs12512631	GC	4	P = 0.0640	**P = 0.0356**
rs16846876	GC	4	P = 0.7397	P = 0.6667
rs17467825	GC	4	P = 0.6168	P = 0.9698
rs222020	GC	4	P = 0.7799	P = 0.5162
rs2282679	GC	4	P = 0.7923	P = 0.9590
rs2298849	GC	4	P = 0.9687	P = 0.4493
rs2298850	GC	4	P = 0.5779	P = 0.9850
rs3755967	GC	4	P = 0.8157	P = 0.9620
rs4588	GC	4	P = 0.5256	P = 0.9767
rs17219315	CYP24A1	20	P = 0.5922	P = 0.7702
rs2244719	CYP24A1	20	P = 0.4368	P = 0.9075
rs229624	CYP24A1	20	P = 0.5013	P = 0.3740
rs2296241	CYP24A1	20	P = 0.4216	P = 0.7995
rs2426496	CYP24A1	20	P = 0.5693	P = 0.7863

rs4809960	CYP24A1	20	P = 0.7945	P = 0.9076
rs6013897	CYP24A1	20	P = 0.0732	P = 2460

Supplementary Table S5. Association of variants of different SNPs selected in the study with overall all microbial abundance using Kruskal-Wallis followed by post hoc Dunn's test (with p-adjusted with Bonferroni).

SNP ID	SNP-variant1	SNP-variant2	p.adj	p.adj.signif
rs12785878	GG	GT	0.0000891	****
	GG	TT	0.447	ns
	GT	TT	0.00909	**
rs2244719	CC	CT	4.38E-08	****
	CC	TT	2.01E-16	****
	CT	TT	6.68E-04	***
rs3755967	AA	AG	7.15E-06	****
	AA	GG	1.64E-10	****
	AG	GG	1.07E-05	****
rs7041	GG	GT	6.03E-08	****
	GG	TT	7.92E-03	**
	GT	TT	3.64E-16	****
rs10500804	GG	GT	0.000732	***
	GG	TT	0.112	ns
	GT	TT	0.221	ns
rs10741657	AA	AG	6.92E-14	****
	AA	GG	7.59E-06	****
	AG	GG	4.95E-11	****
rs12794714	AA	AG	0.000732	***
	AA	GG	0.112	ns
	AG	GG	0.221	ns
rs2282679	AA	AC	1.07E-05	****
	AA	CC	1.64E-10	****
	AC	CC	7.15E-06	****
rs3829251	AA	AG	0.00094	***
	AA	GG	0.000609	***
	AG	GG	1	ns
rs7116978	CC	CT	0.00000013	****
	CC	TT	1	ns

	CT	TT	0.239	ns
rs10766197	AA	AG	0.00000236	****
	AA	GG	0.0143	*
	AG	GG	0.0621	ns
rs1562902	CC	CT	0.0199	*
	CC	TT	0.00296	**
	CT	TT	1	ns
rs229624	CC	CT	1.69E-08	****
	CC	TT	1.24E-27	****
	CT	TT	4.34E-20	****
rs4516035	CC	CT	0.0444	*
	CC	TT	0.0225	*
	CT	TT	1	ns
rs7139166	CC	CG	1	ns
	CC	GG	0.0225	*
	CG	GG	0.0444	*
rs10783219	AA	AT	1.00E+00	ns
	AA	TT	2.93E-42	****
	AT	TT	1.29E-46	****
rs16846876	AA	AT	1	ns
	AA	TT	0.602	ns
	AT	TT	1	ns
rs2296241	AA	AG	0.000175	***
	AA	GG	0.000179	***
	AG	GG	1	ns
rs4588	AA	AC	1.57E-04	***
	AA	CC	1.00E+00	ns
	AC	CC	1.32E-11	****
rs731236	CC	CT	3.13E-04	***
	CC	TT	6.15E-03	**
	CT	TT	1.15E-16	****
rs10877012	GG	GT	4.97E-41	****
	GG	TT	2.79E-01	ns
	GT	TT	8.16E-12	****
rs17219315	AA	AG	0.00000128	****

rs2298849	CC	CT	7.29E-15	****
	CC	TT	6.19E-16	****
	CT	TT	6.49E-01	ns
rs4646536	CC	CT	2.99E-05	****
	CC	TT	9.15E-04	***
	CT	TT	1.69E-31	****
rs11234027	AA	AG	0.000701	***
	AA	GG	0.000878	***
	AG	GG	1	ns
rs17467825	AA	AG	1.32E-11	****
	AA	GG	1.00E+00	ns
	AG	GG	1.57E-04	***
rs2298850	CC	CG	1.32E-11	****
	CC	GG	1.00E+00	ns
	CG	GG	1.57E-04	***
rs4809960	CC	CT	4.81E-11	****
	CC	TT	7.75E-13	****
	CT	TT	1.00E+00	ns
rs1155563	CC	CT	0.0000176	****
	CC	TT	0.00013	***
	CT	TT	0.697	ns
rs1993116	CC	CT	4.95E-11	****
	CC	TT	7.59E-06	****
	CT	TT	6.92E-14	****
rs2426496	GG	GT	2.95E-16	****
	GG	TT	1.00E+00	ns
	GT	TT	3.24E-12	****
rs6013897	AA	AT	5.45E-07	****
	AA	TT	2.83E-01	ns
	AT	TT	2.14E-29	****
rs12512631	CC	CT	0.00197	**
	CC	TT	0.00192	**
	CT	TT	1	ns
rs222020	CC	CT	9.66E-09	****
	CC	TT	1.33E-15	****
	CT	TT	8.87E-08	****
rs703842	CC	CT	2.13E-08	****
	CC	TT	6.98E-06	****
	CT	TT	9.79E-49	****

Supplementary Table S6. Ethnicities of the non-Arab participants in the study.

Non-Arab Ethnicities	Numbers
African	1
Asian	19
European	2
South American	2
American Caucasian	2

Supplementary Table S7. Predictor variables of serum 25-hydroxy vitamin D levels and their coefficient.

**Variable**	**Regression_Coefficient**
Intercept	40.67442513
Age	-0.086363106
BMI_Z_SCORE	-1.202321923
B_F_ratio	-1.211954089
Observed_Species	0.038494135
GenderMale	-4.353997941
EthnictyNon.Arab	0.849253737
Daily_sun_exposureLess.than.1.2.hr	-5.81567709
Daily_sun_exposureMore.than.1.hr	2.161607352
Physical_activityYes.indoor	5.985652302
Physical_activityYes.outdoor	1.692443418
Dairy_product_consumptionYes	10.12865704
rs12512631C.T	-4.709728655
rs12512631T.T	0.468052562
Family_history_of_vitamin_D_deficiencyYes	-0.466009597
Consumption_of_fishMonthly	-2.240606426
Consumption_of_fishNone	-5.205640325
Consumption_of_fishWeekly	0

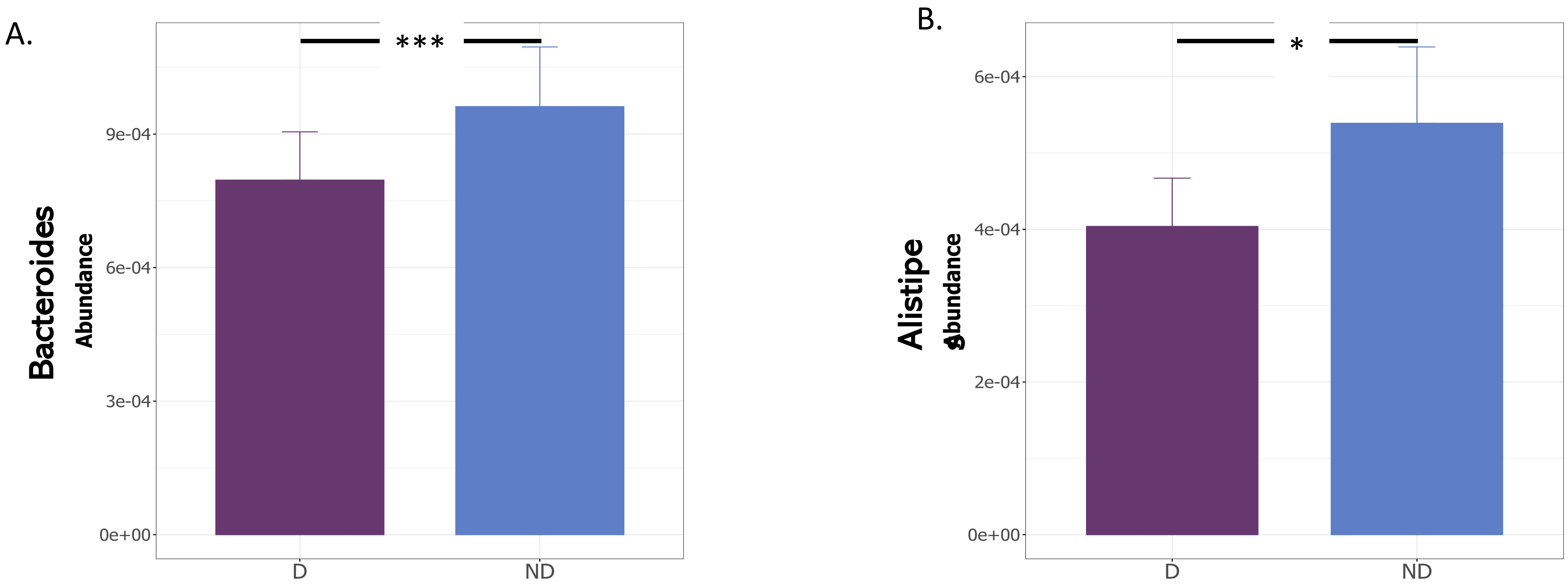

Supplmentary Figure S1

(**A**) Abundance of genus *Bacteroides* (**B**) genus *Alistipes* in Vitamin D deficient(D) and non-deficient groups(ND) groups. (Wilcoxon test with false discovery rate (FDR)-Bonferroni corrected *$P < 0.05$ and ***$P < 0.001$). The figure was generated using (RStudio v 1.4 with R v 4.1).

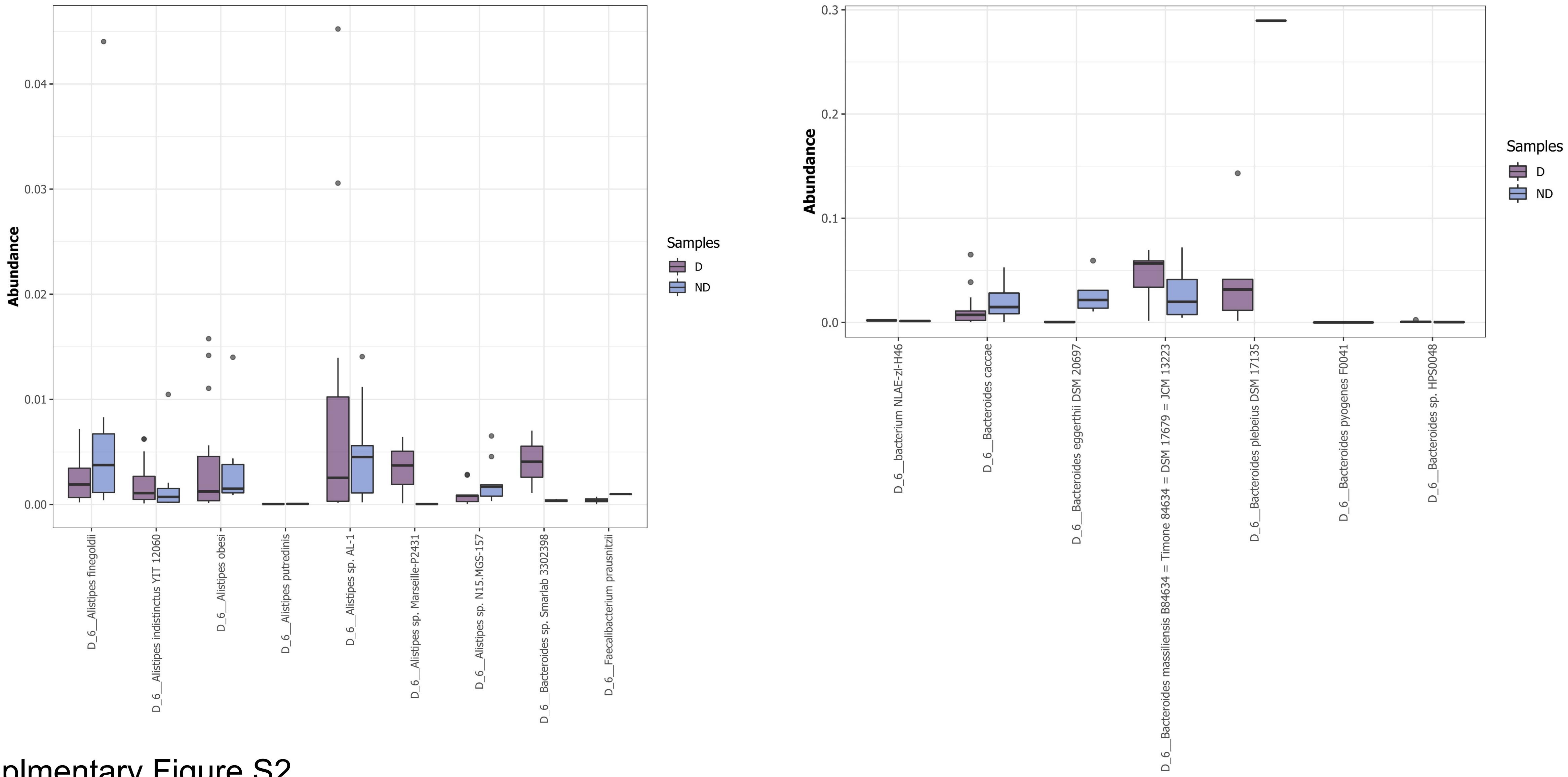

Supplmentary Figure S2

Differences in Abundance of top 20 species between the Vitamin D deficient(D) and non-deficient groups(ND) groups.

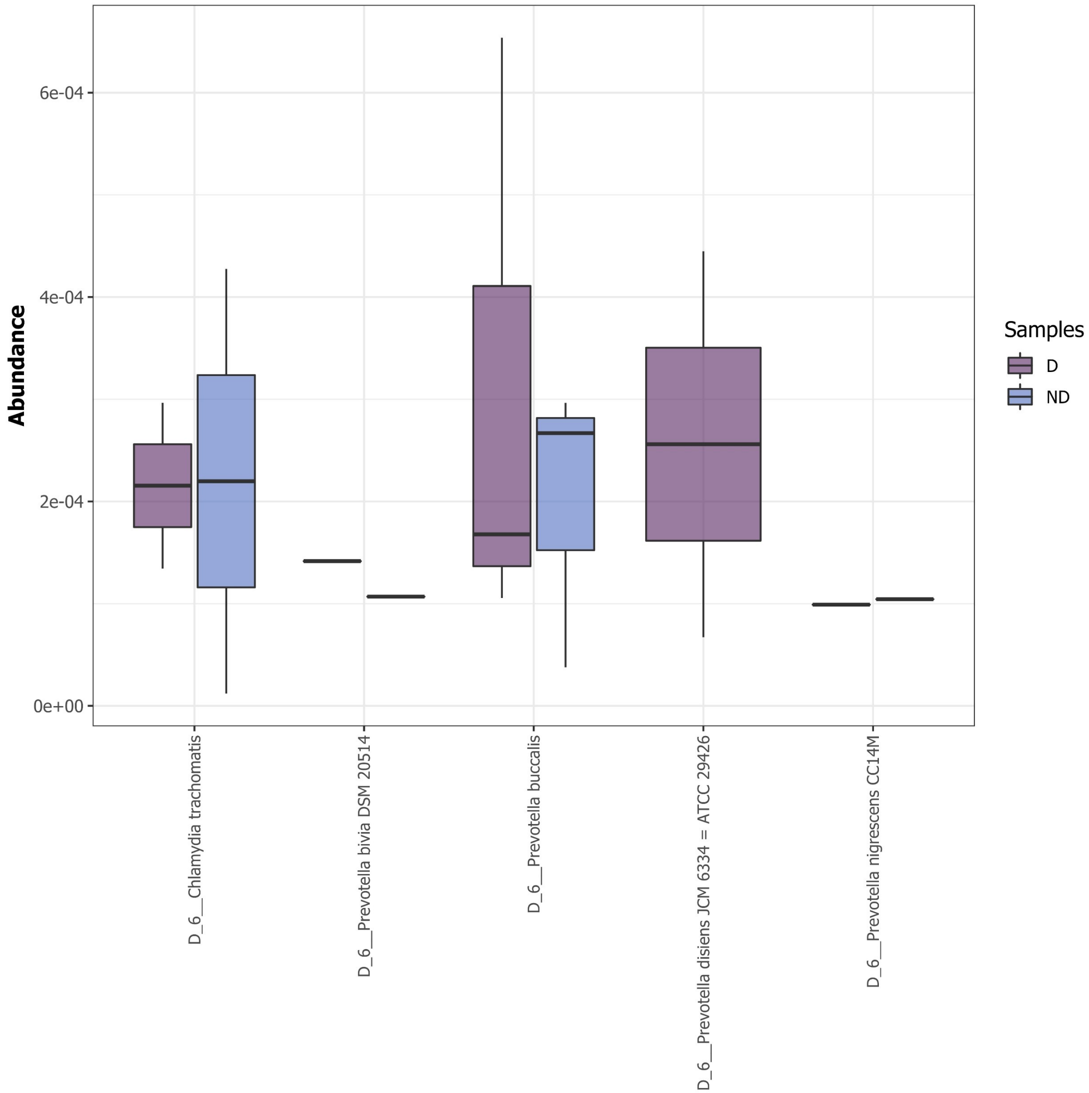

Supplmentary Figure S3

Differences in Abundance of top 20 species between the Vitamin D deficient(D) and non-deficient groups(ND) groups.

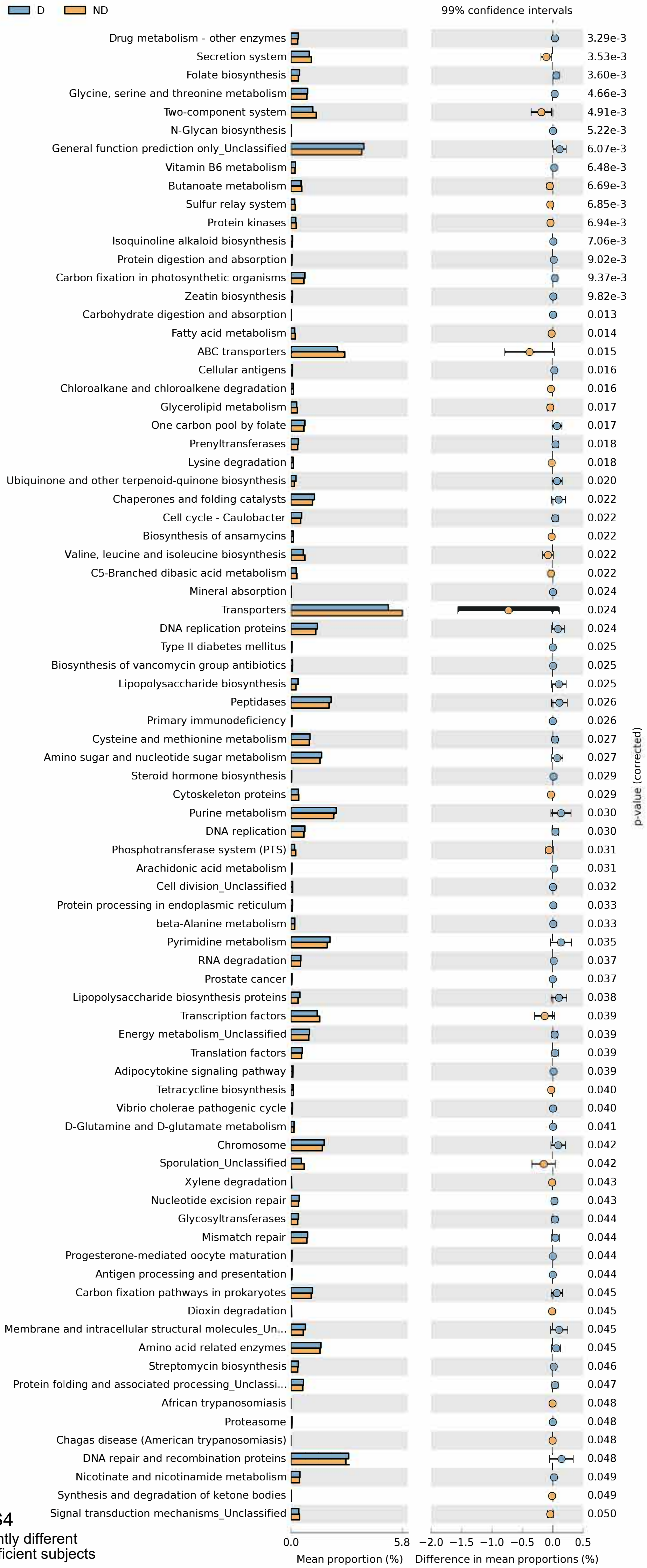

Supplementary Figure S4

Functional pathways significantly different between deficient and non-deficient subjects

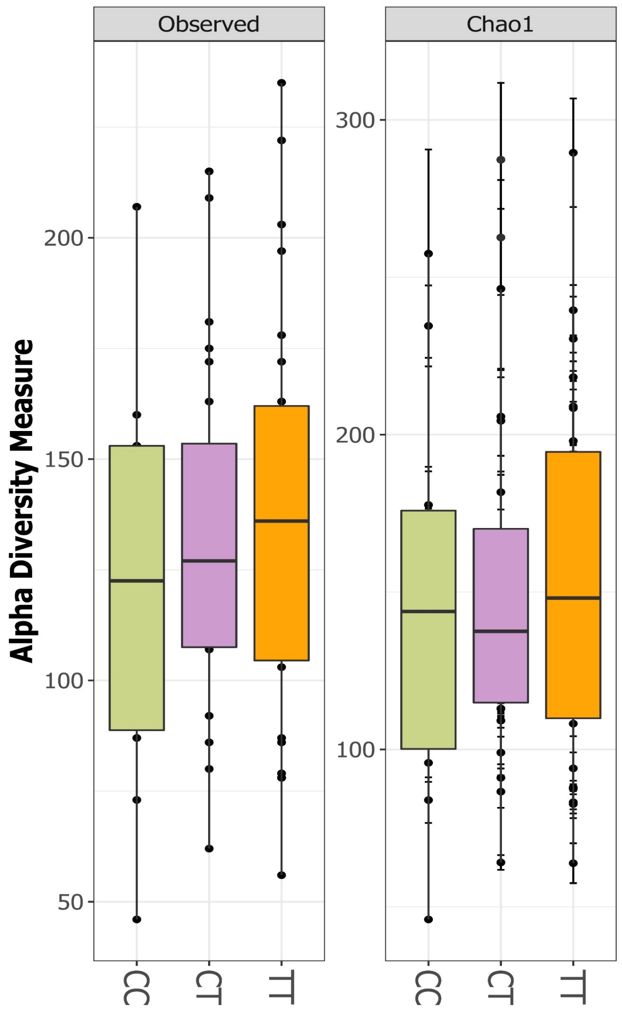

Supplmentary Figure S5

Correlation of alpha diversity measures and the three genotypes of SNP of interest rs12512631 .

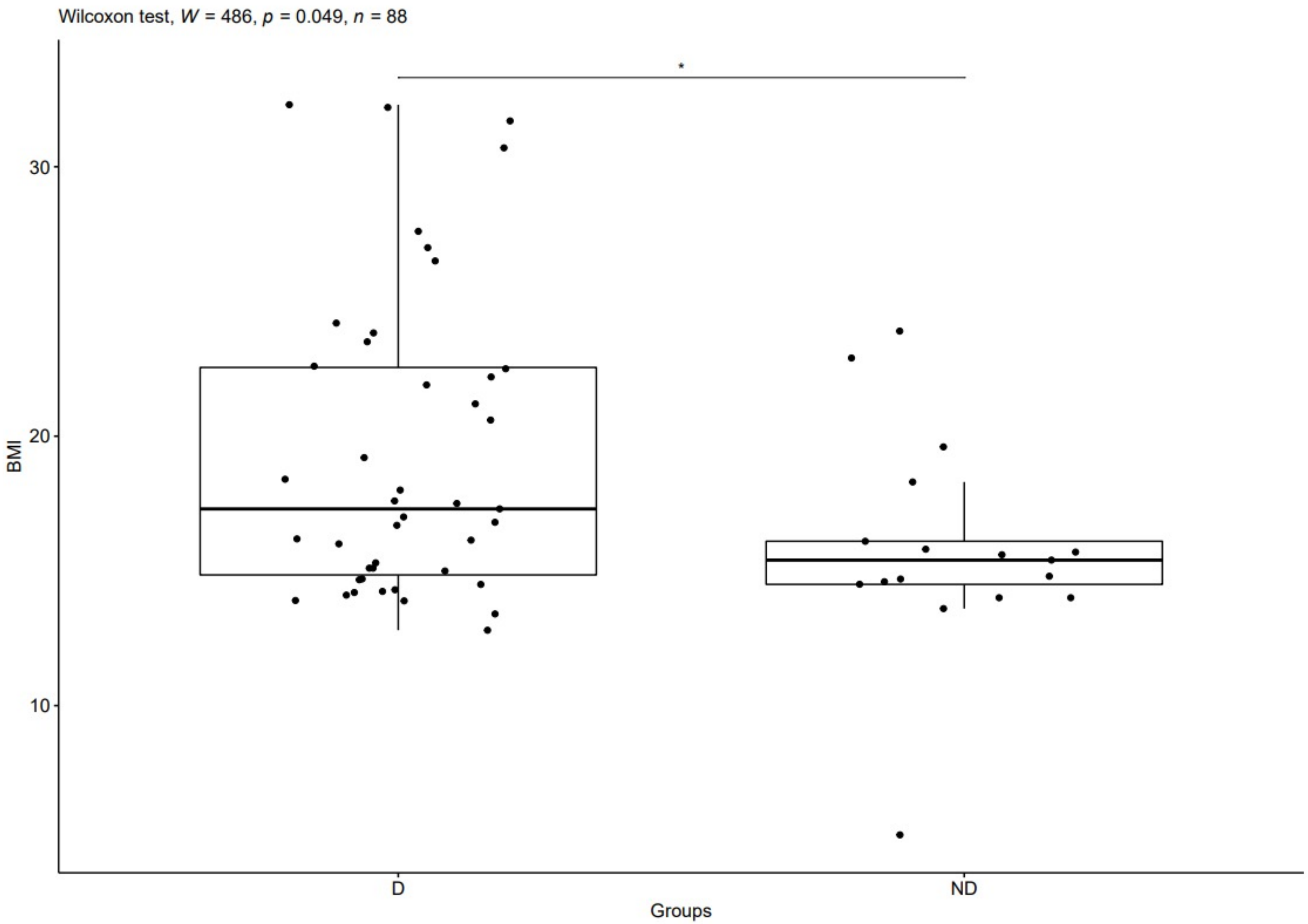

Supplmentary Figure S6

Significant differences in the BMI of Deficient and non-deficient kids.
